# Outcomes of left atrial appendage occlusion with atrial fibrillation ablation: a real-world analysis from the National Inpatient Sample database

**DOI:** 10.1007/s10840-025-02160-2

**Published:** 2025-11-19

**Authors:** Muhammad Zia Khan, Justin Lim Devera, Waleed Alruwaili, Muhammad Abdullah Naveed, Yaseen Maqsood Hakim, William E. Leon, Amanda Nguyen, Siddharth Agarwal, Zain Ul Abideen Asad, Samrina Khan, Karthik Gonuguntla, Daniel Ice, Muhammad Bilal Munir

**Affiliations:** 1 Division of Cardiology, Deborah Heart and Lung Center, Brown Mills, NJ USA; 2 Division of Cardiovascular Medicine, Section of Cardiac Electrophysiology, University of California Davis School of Medicine, 4860 Y St. Suite 2800, Sacramento, CA 95817 USA; 3 Department of Medicine, West Virginia University, Morgantown, WV USA; 4 Department of Cardiology, Dow Medical College, Karachi, Pakistan; 5 Department of Cardiology, Mayo Clinic, Rochester, MN USA; 6 Division of Cardiology, University of Oklahoma, Oklahoma City, OK USA; 7 Department of Medicine, Khyber Medical University, Peshawar, Pakistan; 8 Division of Cardiology, Hartford Hospital Heart and Vascular Institute, Norwich, CT USA

**Keywords:** Atrial fibrillation, Ablation, Left atrial appendage occlusion, Retrospective analysis, Outcomes, Complications

## Abstract

**Background:**

The safety and cost of concomitant atrial fibrillation (AF) ablation and left atrial appendage occlusion (LAAO) procedure remain unknown.

**Objective:**

The study sought to determine real-world outcomes of AF patients who underwent LAAO with ablation.

**Methods:**

The National Inpatient Sample and International Classification of Diseases–Tenth Revision codes were used to identify patients who underwent LAAO with and without AF ablation during the years 2016–2022. The outcomes assessed included procedural complications and resource utilization.

**Results:**

LAAO with AF ablation was associated with a higher rate of overall (odds ratio [OR] 1.54, 95% confidence interval [CI] 1.37–1.74), major complications (OR 1.38, 95% CI 1.18–1.60), non-home discharge (OR 1.55, 95% CI 1.23–1.96), prolonged length of stay > 1 day (OR 3.21, 95% CI 2.92–3.52), and increased hospitalization costs as defined by median cost > $25,926 (OR 19.42, 95% CI 16.21–23.25) when compared to LAAO alone after adjustment for potential confounding variables. Patients who underwent LAAO with ablation were also more likely to receive pacemaker implantation (3.6% vs 0.2%, *P* < 0.001) and experience acute kidney injury (3.9% vs 2.1%, *P* < 0.001) and non-ST elevation myocardial infarction (3.7% vs 1.5%, *P* < 0.001).

**Conclusion:**

In a large, contemporary, real-world study of LAAO procedures in the USA, concomitant AF ablation was associated with a higher rate of overall and major complications and an increased resource utilization.

**Graphical Abstract:**

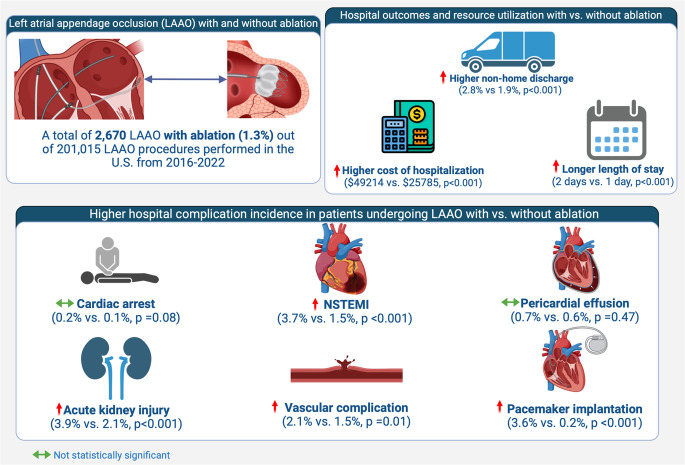

## Introduction

Catheter ablation of atrial fibrillation (AF) is effective in reducing arrhythmia burden although it is associated with ongoing risk of stroke and thromboembolism, especially in the first several weeks post-ablation [1, 2]. Existing guidelines therefore recommend oral anticoagulation for at least 3 months or longer based on stroke risk after AF ablation [3]. Percutaneous left atrial appendage occlusion (LAAO) has become an alternative treatment option for stroke prophylaxis, with the first device approved by the Food and Drug Administration (FDA) in 2015 [4, 5]. Currently, the data on safety of combined AF ablation with LAAO procedure is limited, although the recently published OPTION (left atrial appendage closure after ablation for atrial fibrillation) trial demonstrated that LAAO with ablation is associated with a lower risk of major bleeding and is non-inferior to oral anticoagulation in terms of all-cause mortality, stroke, and systemic embolism at 36 months [6]. We sought to examine real-world data on outcomes of AF patients who underwent LAAO with ablation in the USA in the contemporary era.

## Methods

### Data source

Data from the National Inpatient Sample (NIS) were used for this study [7]. We analyzed the NIS database from years 2016–2022 for cases of LAAO device placement performed with and without AF ablation. The NIS is a large hospital-based administrative database which was made possible by a federal-state-industry sponsorship sponsored by the Agency for Healthcare Research and Quality. The NIS can be used for computing national estimates of in-hospital healthcare utilization, costs, and outcomes. Of note, NIS does not adequately capture procedures that are mainly conducted on outpatient basis. The data are de-identified; therefore, the need for informed consent and Institutional Review Board approval is waived. The NIS adheres to the 2013 Declaration of Helsinki for the conduct of human research.

### Study population

LAAO device placements were identified using International Classification of Diseases–Tenth Revision–Clinical Modification (ICD-10-CM) code of 02L73DK. Patients younger than 18 years of age and those with missing demographic data were excluded. The subset of patients who had AF ablation performed during the same hospital encounter were identified using ICD-10-CM code of 02583ZZ. The outcomes of patients who underwent LAAO alone versus those who underwent both LAAO and AF ablation were then compared. The outcomes of interest were similar to those identified as major adverse events in the National Cardiovascular Data Registry (NCDR) LAAO Registry, as well as earlier studies on patients undergoing LAAO [8–10]. Patients with mechanical ventilation were identified as those who were unable to be extubated after the procedure and require artificial ventilation for greater than 36 h.

Baseline characteristics, complications, and inpatient outcomes including mortality, length of stay, discharge disposition, and hospitalization costs were analyzed. For computation of hospitalization costs, the cost-to-charge ratio files based on Centers for Medicare and Medicaid Services reimbursement and provided by the Healthcare Cost and Utilization Project were applied to the total hospital charges.

### Statistical analysis

Descriptive statistics are presented as frequency and percentage for categorical variables and as median and interquartile range for continuous variables. Baseline characteristics were compared using a Pearson chi-square test and Fisher exact test for categorical variables and the Kruskal–Wallis *H* test for continuous variables. For crude comparison of procedural complications and in-hospital outcomes among the study groups, the Pearson chi-square test was used.

Logistic regression was performed to estimate odds ratios (OR) with 95% confidence intervals (CI) of major complications, non-home discharges, length of stay > 1-day, and median hospitalization cost > $25,926 in patients who underwent LAAO and AF ablation compared to LAAO alone. To assess the independent association of combined LAAO and AF ablation with these outcomes, a single-step multivariable logistic regression model was used to adjust for potential confounders. Age, sex, race/ethnicity, income, insurance status, and selected Elixhauser comorbidities were used for adjusted analysis. All these covariates were identified based on prior literature, bivariate analysis, and the authors’ best clinical judgment [11, 12]. A *P* value of < 0.05 was considered statistically significant. All statistical analyses were performed using SPSS version 26 (IBM Corporation) and R version 3.6 (R Foundation for Statistical Computing). Because of the complex survey design of the NIS, sample weights, strata, and clusters were applied to raw data to generate national estimates [7].

## Results

A total of 201,015 cases of LAAO device placement were analyzed after excluding for missing demographics. Out of these, approximately 2670 (1.3%) LAAO device placements were associated with AF ablation. Median age was less in patients who underwent LAAO with ablation compared to LAAO alone (median age 74 vs 77, *P* < 0.001), and there was no significant difference in both groups based on sex (Table 1). LAAO with AF ablation is associated with a lower prevalence of comorbidities such as cerebrovascular disorder (4.5% vs 6.8%, *P* < 0.01), congestive heart failure (33% vs 34.6%, *P* < 0.01), chronic pulmonary disease (18% vs 21.5%, *P* < 0.01), coronary artery disease (14.6% vs 17.4%, *P* < 0.01), diabetes (14.6% vs 17.4%, *P* < 0.01), chronic kidney disease (21.2% vs 23.7%, *P* < 0.01), hypertension (83.9% vs 87.1%, *P* < 0.01), and peripheral vascular disease (6% vs 8.7%, *P* < 0.01).

**Table 1:** Baseline characteristics of study group

Variable no. (%)	LAAO without ablation *N* = 198,345 (98.7%)	LAAO with ablation *N* = 2670 (1.3%)	*p*-value
Age (median [IQR]) years	77 (72–82)	74 (68–79)	< 0.001
Females	82,455 (41.60%)	1055 (39.5)	0.32
Age < 65	13,010 (6.60%)	330 (12.4)	< 0.001
65–74	63,685 (32.10%)	1060 (39.7)	
≥ 75	111,995 (56.50%)	1165 (43.6)	
Race			
White	170,230 (88.70%)	2285 (87.2%)	< 0.001
Black	7510 (3.90%)	110 (4.2%)	
Hispanic	8120 (4.20%)	165 (6.3%)	
Asian or Pacific Islander	2470 (1.30%)	30 (1.1%)	
Native American	615 (0.30%)	0 (0.0%)	
Other	2885 (1.50%)	30 (1.1%)	
Comorbidities			
Deficiency anemia	7175 (3.60%)	135 (5.1%)	< 0.001
Cerebrovascular disorders	13,470 (6.80%)	120 (4.5%)	< 0.001
Congestive heart failure	68,635 (34.60%)	880 (33.0%)	< 0.001
Chronic pulmonary disease	42,720 (21.50%)	480 (18.0%)	< 0.001
Coronary artery disease	34,485 (17.40%)	390 (14.6%)	< 0.001
Diabetes	34,485 (17.40%)	390 (14.6%)	< 0.001
Chronic kidney disease	47,080 (23.70%)	565 (21.2%)	0.002
Hypertension	172,720 (87.10%)	2240 (83.9%)	< 0.001
Liver disease	5740 (2.90%)	80 (3.0%)	0.754
Obesity	35,610 (18.00%)	520 (19.5%)	0.042
Peripheral vascular disorders	17,185 (8.70%)	160 (6.0%)	< 0.001
Hypothyroidism	34,925 (17.60%)	490 (18.4%)	0.316
Smoking status	8745 (4.40%)	170 (6.4%)	< 0.001
Median income			
0–25th	43,335 (22.10%)	630 (24.0%)	< 0.001
26–50th	52,065 (26.60%)	610 (23.3%)	
51–75th	53,225 (27.20%)	620 (23.7%)	
76–100th	47,285 (24.10%)	760 (29.0%)	
Insurance			
Medicare	175,195 (88.40%)	2275 (85.2%)	< 0.001
Medicaid	2395 (1.20%)	30 (1.1%)	
Private insurance	16,700 (8.40%)	305 (11.4%)	
Self-pay	600 (0.30%)	15 (0.6%)	
No charge	50 (0.00%)	0 (0.0%)	
Other	3230 (1.60%)	45 (1.7%)	
Region			
Northeast	20,315 (10.20%)	355 (13.3%)	< 0.001
Midwest	34,225 (17.30%)	440 (16.5%)	
South	58,605 (29.50%)	905 (33.9%)	
West	31,015 (15.60%)	325 (12.2%)	

Values are *n* (%) or median (IQR)

*IQR* interquartile range, *LAAO* left atrial appendage occlusion

In patients undergoing LAAO with ablation, there was a higher incidence of overall complications (12% vs 8.6%, *P* < 0.001) and major complications (defined as a composite of cardiac arrest/cardiopulmonary resuscitation (CPR), ischemic stroke, hemorrhagic stroke, transient ischemic attack, arterial embolism, myocardial infarction, major bleeding, pericardial effusion requiring intervention, and peripheral vascular complications) (5.8% vs 2.5%, *P* < 0.001) (Table 2). Additionally, LAAO with ablation was associated with higher incidence of pacemaker implantation (3.6% vs 0.2%, *P* < 0.001) and acute kidney injury (3.9% vs 2.1%, *P* < 0.001). Non-ST elevation myocardial infarction (NSTEMI) was more prevalent in the LAAO with ablation group (3.7% vs 1.5%, *P* < 0.001), and there was no significant difference in terms of STEMI. Pericarditis occurred more frequently in LAAO with ablation group (0.4% vs 0.1%, *P* < 0.001), although there were no differences in pericardial effusion requiring intervention (0.7% vs 0.6%, *P* = 0.474) or cardiac arrest requiring CPR (0.2% vs 0.1%, *P* = 0.089). Less than 11 data were not reported as per recommendations of the data source

**Table 2:** Complications in patients undergoing percutaneous LAAO

Variables no. (%)	LAAO without ablation *N* = 198,345 (98.7%)	LAAO with ablation *N* = 2670 (1.3%)	*p*-value
Overall complications (%)	16,995 (8.6)	320 (12.0)	< 0.001
Major complications (%)^*^	10,840 (5.5)	185 (6.9)	0.001
Any cardiovascular event/complication	4970 (2.5)	155 (5.8)	< 0.001
Percutaneous coronary intervention	20,445 (10.3)	265 (9.9)	0.518
Cardiac arrest/CPR procedure code	175 (0.1)	< 11(0.2)	0.089
Pacemaker implantation	460 (0.2)	95 (3.6)	< 0.001
STEMI	55 (0.0)	0 (0.0)	0.389
NSTEMI (type I or II)	2940 (1.5)	100 (3.7)	< 0.001
Pericardial effusion requiring intervention	1265 (0.6)	20 (0.7)	0.474
Tamponade	855 (0.4)	15 (0.6)	0.307
Pericarditis	175 (0.1)	< 11 (0.4)	< 0.001
Cardiogenic shock	360 (0.2)	15 (0.6)	< 0.001
Any systemic complication	230 (0.1)	< 11 (0.2)	0.284
Anaphylaxis	50 (0.0)	0 (0.0)	0.412
Arterial embolism	110 (0.1)	0 (0.0)	0.224
Septic shock	75 (0.0)	< 11 (0.2)	< 0.001
Any peripheral vascular complication	2945 (1.5)	55 (2.1)	0.015
AV fistula	175 (0.1)	< 11 (0.4)	< 0.001
Pseudoaneurysm	515 (0.3)	0 (0.0)	0.008
Retroperitoneal bleeding	945 (0.5)	20 (0.7)	0.043
Dissection	90 (0.0)	0 (0.0)	0.271
Venous thromboembolism	440 (0.2)	< 11(0.4)	0.097
Any neurological complication	1385 (0.7)	15 (0.6)	0.4
Hemorrhagic stroke	500 (0.3)	15 (0.6)	0.002
Ischemic stroke	485 (0.2)	< 11 (0.2)	0.551
TIA	435 (0.2)	0 (0.0)	0.015
Any GI or hematological complication/bleeding	7020 (3.5)	75 (2.8)	0.042
GI bleeding	4545 (2.3)	30 (1.1)	< 0.001
Bleeding during the procedure	170 (0.1)	0 (0.0)	0.130
Need for blood transfusion	2605 (1.3)	45 (1.7)	0.094
Any pulmonary complications	4000 (2.0)	85 (3.2)	< 0.001
Respiratory failure	1865 (0.9)	20 (0.7)	0.309
Pneumothorax	60 (0.0)	0 (0.0)	0.369
Pleural effusion	770 (0.4)	< 11(0.4)	0.910
Pneumonia bacterial	410 (0.2)	15 (0.6)	< 0.001
Mechanical ventilation (> 36 h)	135 (0.1)	< 11 (0.2)	0.020
Acute kidney injury	4110 (2.1)	105 (3.9)	< 0.001

Values are *n* (%). *Defined as composite of cardiac arrest/CPR, ischemic stroke, hemorrhagic stroke, TIA, arterial embolism, myocardial infarction (STEMI or NSTEMI), major bleeding, pericardial effusion requiring intervention, and peripheral vascular complications

Less than 11 data were not reported as per recommendations of the data source

*AV* arteriovenous, *CPR* cardiopulmonary resuscitation, *GI* gastrointestinal, *LAAO* left atrial appendage occlusion, *NSTEMI* non-ST elevation myocardial infarction, *STEMI* ST-elevation myocardial infarction, *TIA* transient ischemic attack

LAAO with ablation was associated with higher rate of non-home discharge (2.8% vs 1.9%, *P* = 0.001), longer length of stay (interquartile range 1–2 days vs 1 day, *P* < 0.001), and increased cost of hospitalization (median $49,214.09 vs $25,785.74, *P* < 0.001). There was no difference in in-hospital death (0.0% vs 0.1%, *P* < 0.064) between the two groups (Table 3).

**Table 3:** Hospital outcomes and resource utilization in patients undergoing LAAO

Variables no. (%)	LAAO without ablation *N* = 198,345 (98.7%)	LAAO with ablation *N* = 2670 (1.3%)	*p*-value
Died at discharge	255 (0.1)	0 (0.0)	0.064
Home/routine/self-care	194,210 (98.1)	2595 (97.2)	0.001
Non-home discharges	3845 (1.9)	75 (2.8)	
Length of stay, days	1 (1–1)	1 (1–2)	< 0.001
Cost of hospitalization, $	25,785.74 (20,145.45–32,749.96)	49,214.09 (37,995.79–63,567.67)	< 0.001

Values are *n* (%) or median (IQR)

*IQR* interquartile range, *LAAO* left atrial appendage occlusion

**Fig. 1:**
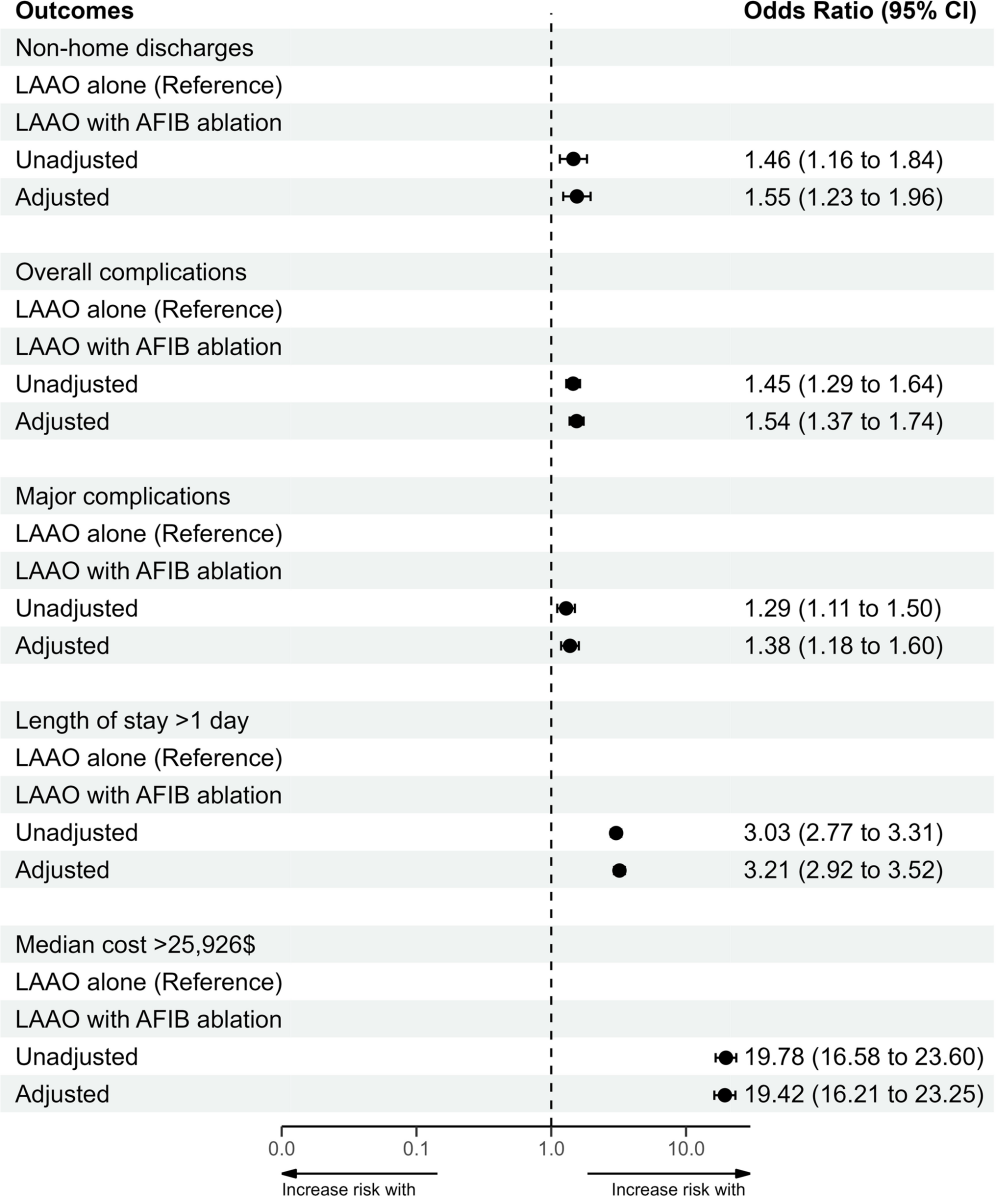
Adjusted associations of LAAO with AF ablation with outcomes of non-home discharges, overall complications, major complications, prolonged length of stay, and hospitalization cost. AF = atrial fibrillation; CI = confidence interval; LAAO = left atrial appendage occlusion

Multivariable models adjusting for potential confounders were created to assess the independent association of LAAO and AF ablation with adverse outcomes and using LAAO only as a reference (Fig. 1). After adjustment for potential confounding variables, LAAO with AF ablation was associated with a higher rate of overall complications (OR 1.54, 95% CI 1.37–1.74), major complications (OR 1.38, 95% CI 1.18–1.60), prolonged length of stay > 1 day (OR 3.21, 95% CI 2.92–3.52), and increased hospitalization cost as defined by the median cost > $25,926 (OR 19.42, 95% CI 16.21–23.25).

## Discussion

In this contemporary, real-world study of LAAO cases in the USA, we report several important findings: (1) Approximately 1.3% (2670 of 201,015) of such implantations were performed with AF ablation from years 2016–2022. (2) LAAO with ablation was associated with a higher incidence of several in-hospital complications and an increased resource utilization in the crude analysis. (3) LAAO with ablation was associated with a higher rate of overall and major complications, increased likelihood of non-home discharge, near-double cost of hospitalization, and longer length of stay in the adjusted analysis. It is not surprising that the addition of ablation to LAAO costs more than LAAO alone, and the increased upfront cost of a dual procedure should be weighed against the potential long-term cost-savings in achieving rhythm control.

Anticoagulation after catheter ablation of AF remains an area of active research, with interest in minimizing bleeding risk associated with oral anticoagulation while also reducing the incidence of ischemic events [13]. Current guidelines recommend postprocedural anticoagulation for at least 3 months, with longer duration determined by individualized stroke risk [3]. Recently, the OPTION (left atrial appendage closure after ablation for atrial fibrillation) trial compared LAAO to anticoagulation in patients after AF ablation and found that LAAO was associated with a lower risk of non-procedure-related bleeding (8.5% vs 18.1%, *P* < 0.001) and was non-inferior to oral anticoagulation in terms of a composite end-point of death from any cause, stroke, or systemic embolism at 36 months (5.3% vs 5.8%, *P* < 0.001) [6]. Of note, most patients in the OPTION trial underwent sequential LAAO, with about 60% receiving device implant 90–180 days after ablation, while the rest underwent ablation with concomitant LAAO [6]. Therefore, our study provides a unique perspective on outcomes of patients undergoing concomitant procedures, who are not represented by the majority of patients in the OPTION trial [6].

Our study provides real-world data that reveal important differences in short-term outcomes and resource utilization in patients undergoing AF ablation with and without LAAO. Interestingly, in contrast to the findings of our study, the OPTION trial found a lower incidence of acute myocardial infarction (9/808 [1.1%] vs 7/797 [0.9%]) and acute kidney injury (13/797 [1.6%] vs 15/803 [1.9%]) in the ablation with LAAO versus ablation with oral anticoagulation group, respectively. These discrepancies may be due in part to differences in patient population; notably, the OPTION trial excluded patients with myocardial infarction within 90 days and left ventricular ejection fraction < 30%, thereby creating the potential for selection bias and hence such outcomes [6]. In contrast, our study included all patients undergoing LAAO without exclusion of comorbidities and is therefore more representative of patients across the spectrum of cardiovascular disease.

Our study identified a nearly twofold increased incidence of acute kidney injury after LAAO and AF ablation compared to LAAO alone. This might be explained by an increased use of iodine-based contrast that are usually employed for LAAO device sizing and implantation hence leading to contrast-induced nephropathy [14]. Additionally, longer procedure time can increase the risk of renal hypoperfusion secondary to the vasodilatory effects of anesthesia and direct kidney injury can ensue from the inflammatory effects of surgery and hemolysis [15, 16].

Non-ST elevation myocardial infarction was diagnosed in more than twice as many patients undergoing LAAO with ablation than in those with LAAO alone. The likely etiologies for this are less certain, although it is likely that most cases of NSTEMI were secondary to supply/demand mismatch (i.e., type 2 myocardial infarction) and not acute thrombosis (i.e., type 1), given there was no significant difference in the rate of percutaneous coronary intervention (PCI) between these two groups. Additionally, troponin elevation could have also been due to acute myocardial injury from ablation itself [17–19]. Nonetheless, AF ablation has been reported to cause an inflammatory state which can be pro-thrombotic, especially within the first 2 weeks post-ablation [20, 21].

There was a higher rate of pacemaker implantation in patients who underwent LAAO with ablation when compared to LAAO device implantations only. These findings are congruent with a retrospective analysis by Deshmukh et al., who found that pacemaker implantation occurred in about 1/28 (3.6%) patients within 1 year after AF ablation [22]. The authors also found no significant difference in the risk of pacemaker implantation in patients who received catheter ablation and those who received cardioversion, suggesting that the need for pacing is secondary to underlying pathological substrate and not the ablation itself [22]. Therefore, the greater incidence of pacemaker implantation after combined LAAO with AF ablation in our cohort could likely be explained by the unmasking of sinus node dysfunction or other conduction system disease upon restoration of sinus rhythm after ablation and in settings of prolonged anesthesia time (when concomitant LAAO implantation were performed). Fortunately, there were no significant differences in terms of pericardial effusion requiring intervention and cardiac tamponade, which remains the most significant complication after AF ablation and LAAO device implantations [23].

## Limitations

The results of our current study should be interpreted in the context of the following limitations. First, the NIS relies on ICD-10-CM codes for disease and procedure identification, which may be subject to errors; however, the NIS uses a rigorous data quality control program to minimize miscoding and ensures integrity of data [7]. Second, long-term outcomes cannot be ascertained from the present dataset, as the NIS includes index admission data only. Third, there are no data available on procedural steps involved with LAAO placement and AF ablation such as the type of device implanted (e.g., WATCHMAN 2.5 or FLX [Boston Scientific] or Amulet [Abbott], which had been approved by the FDA during the inclusion period), energy source for ablation (i.e., radiofrequency, cryoablation, or pulsed field ablation), utilization of contrast, and operator experience [5]. Fourth, the NIS is specific to inpatient admissions and does not provide information on outpatient encounters or post-hospitalization anticoagulation/antiplatelet prescription patterns. Fifth, the NIS is not equipped to delineate the granular mechanisms of outcomes in the study group, nor to provide the explicit indications for procedures such as pacemaker implantation. Although we have utilized a rigorous regression analysis for delineation of adjusted outcomes, residual confounding due to unmeasured variables is not excluded due to retrospective nature of NIS dataset. Sixth, the generalizability of our findings is limited because the NIS only captures inpatients who are likely to be at higher baseline risk of complications, thus potentially explaining why the overall complication rate in both groups in our study is higher than other large real-world data sets [24].

Finally, it is difficult to ascertain the extent to which each complication is secondary to AF ablation versus LAAO. While AF ablation does require multiple vascular access and general anesthesia in most centers, increasing risk of complications, the NCDR demonstrated that in patients undergoing AF ablation, there was low prevalence of major complications (0.9%), pericardial effusion requiring intervention (0.44%), access site bleeding requiring transfusion (0.15%), major vascular complications (0.08%), and stroke/transient ischemic attack (0.16%) [25]. Additionally, only 11.8% of patients were hospitalized for more than 1 day [25]. The overall safety of AF ablation is further supported by the ESC-EORP European Heart Rhythm Association Atrial Fibrillation Ablation Long-Term registry, which reported low prevalence of in-hospital complications after ablation: major cardiovascular (4.1%), cardiac perforation (1.3%), peripheral/vascular (1.3%), and stroke (0.1%) [26].

Conversely, serious pericardial effusion and major bleeding were the most frequent adverse events in patients undergoing percutaneous LAAO with WATCHMAN 2.5 in the PROTECT AF trial, which compared the device group to warfarin for stroke prevention in patients with AF [27, 28]. The incidence of serious pericardial effusion was 4.8%, major bleeding 4.8%, procedure-related ischemic stroke 1.3%, and hemorrhagic stroke 0.6% in the device group [28]. Our study population included patients with WATCHMAN 2.5 and the newer FLX model, and it is worth noting that the latter is associated with lower rates of major bleeding (3.1%), ischemic stroke (0.23%), and pericardial effusion requiring intervention (0.50%) in the NCDR’s LAAO Registry at 45 days post-procedure [29].

## Conclusion

Our findings provide insight into the real-world outcomes during hospitalization of patients undergoing LAAO with AF catheter ablation. The performance of both procedures may be intended to avoid the risks of major bleeding with oral anticoagulation after ablation, as explored in the recent OPTION trial. We found that LAAO with ablation was significantly associated with several in-hospital complications including NSTEMI (likely type II given no between-group difference in PCI), pericarditis, cardiogenic shock, pacemaker implantation, peripheral vascular complications, hemorrhagic stroke, TIA, pneumonia, and acute kidney injury. Additionally, LAAO with ablation was associated with longer length of stay and near-double cost of hospitalization. Further studies are needed to elucidate the short-term safety and cost-effectiveness of LAAO with catheter ablation of AF.
